# Foot Plantar Pressure Abnormalities in Near Adulthood Patients with Type 1 Diabetes

**DOI:** 10.3390/biomedicines11112901

**Published:** 2023-10-26

**Authors:** Marta Wysocka-Mincewicz, Ewa Szczerbik, Maria Mazur, Magdalena Grabik, Małgorzata Kalinowska, Małgorzata Syczewska

**Affiliations:** 1 Clinic of Endocrinology and Diabetology, Children’s Memorial Health Institute, 04-730 Warsaw, Poland; m.mazur@ipczd.pl (M.M.); m.grabik@ipczd.pl (M.G.); 2 Kinesiology Lab, Children’s Memorial Health Institute, 04-730 Warsaw, Poland; e.szczerbik@ipczd.pl (E.S.); m.kalinowska@ipczd.pl (M.K.); m.syczewska@ipczd.pl (M.S.)

**Keywords:** type 1 diabetes, children, diabetic foot complications, pedography, peak pressure, force integral pressure, foot contact area, foot plantar pressure

## Abstract

Increased ulcer risk diminishes the quality of life in diabetes. This study assessed abnormalities in foot plantar pressure distribution in adolescents with T1D to detect early signs of ulcer risk. A total of 102 T1D patients, without diabetic neuropathy, were included (mean age 17.8 years, mean diabetes duration 7.4 year). Pedography was captured using Novel emed. Data from the study group were compared with reference data. The study revealed a statistically significant reduced foot contact area in both feet in the entire foot and under the head of the fifth metatarsal bone and the second toe. In both feet, the peak pressure was increased under the entire foot, hindfoot, midfoot, first metatarsal head, big toe, and second toe. There was no statistically significant difference in peak pressure. The mean plantar pressure rating was statistically significantly increased in both feet across the entire sole, in the hindfoot, midfoot, and first metatarsal head. T1D patients of age near adulthood without neuropathy have increased values in mean pressure and reduced contact area, pointing to the need of monitoring and preventive measures. These results point to the need of further research and analysis which should include various risk factor such as foot anatomy, body posture, or certain metabolic factors.

## 1. Introduction

Diabetes is a chronic metabolic disease with many late complications, which burdens patients’ lives and significantly diminishes their quality of life, leading to disability and mortality. One of the most burdensome complications is symmetrical peripheral diabetic neuropathy (SPDN), which causes foot ulcers, often leading to amputations. Children are the group of patients with type 1 diabetes (T1D) who suffer from T1D almost all their lifespans. Therefore, the preventive measures and assessment of the risk factors with possibility to detect early signs are especially important in this group. The lack of effective, causal treatment for SPDN highlights the importance of common and accessible early diagnosis to prevent secondary complications and introduce prophylaxis. The key factors for the risk of foot ulceration include peripheral neuropathy, foot deformity, peripheral vascular disease, previous foot ulceration, and previous amputation of part of the foot or leg [1].

Proper biomechanics of the foot (both anatomy and function) is responsible for preserving the normal (i.e., characteristic for healthy subjects) distribution of plantar pressure, and maintaining proper body posture [2,3]. Zulkifli and Loh [4] revealed that independently of assessment methods, under identical conditions, the pressure range of a healthy foot differs from the unhealthy one. Altered plantar pressure distribution can depend on age, poor fitness status, and illnesses in which various foot and body deformities can arise [1,3].

Pedography evaluates plantar pressure distribution on the foot while standing or walking [5]. It is a low-cost and non-invasive method which can be used in musculoskeletal system diagnostics. Its most common application is the assessment of the risk of ulceration in adult patients with type 2 diabetes [6,7,8,9]. Increased local plantar pressure in certain areas of the foot in combination with diabetic angiopathy and neuropathy can lead to diabetic foot syndrome [1,10,11]. The described abnormalities of plantar pressure distribution in diabetic patients include, especially, the forefoot and its areas under the head of II and III metatarsal bones [6,12], and these are the spots where ulcers are most often formed [6]. Using plantar pressure data, other parameters, analyzed in studies of diabetes, are calculated: force, contact area, and integral variables (describing the pressure or force in time). Waldecker established the foot ulcer predictors, based on pedography, with a sensitivity of 95% and a specificity of 90%. These predictors are the pressure time integral in the metatarsal bone 2 (MH2), force in the second toe (2Toe), the force time integral in the big toe, peak pressure in metatarsal bone 4 (MH4), pressure time integral in MH4, peak pressure in the big toe, peak pressure in metatarsal bone 5 (MH5), and force in MH4 [13].

However, most studies applying plantar pressure distribution focus on assessing adults with type 2 diabetes, where the disease develops most commonly at a mature age and is asymptomatic for a long time. To our knowledge there is not any pedographic study of children with T1D. Of course, in the mature age of patients with T1D diabetic foot is the sum of the overlapping influence of diabetes, all traumas experienced during the lifetime, improper shoes, and many other factors and comorbidities which could additionally disable the human locomotor system. In pediatric patients, late diabetes complications are very rare, and in the Children’s Memorial Health Institute in Warsaw, so far there has been no case of any diabetic foot syndrome, no chronic kidney disease and no established diabetic retinopathy reported. Due to young age, and the most physiological diabetes treatment using an insulin pump, frequently with continuous glucose monitoring systems, in a pediatric center, neurological and angiopathic disorders contributing to the development of complications are extremely rare. Sometimes, just a few years after the transfer to an adult diabetic center patients develop signs and symptoms of advanced peripheral neuropathy, including in some cases Charcot foot. Foot ulcer risk is estimated at the level between 15 and 25% throughout a lifetime, despite the type of diabetes; thus, approximately a quarter of T1D patients are endangered by this complication [8].

Despite medical care, monitoring, and access to the best diabetes therapies, many young T1D patients have poor metabolic control. Moreover, type 1 diabetes develops in children quite often at a very young age. During childhood and adolescence, the body grows and shapes, and this also applies to the feet. If this process is altered by metabolic disturbances, it can lead to the development of abnormalities in the musculoskeletal system, increasing the risk of ulcer appearance.

This analysis is intended to examine if in adolescents with T1D, there are abnormalities in foot plantar pressure distribution, and to identify the risk group for early foot ulceration development. Such identification will allow for the design of correct prophylaxis and foot care, such as customized orthopedic shoe insoles, customized footwear, or toe orthoses to prevent this serious complication of diabetes in the future [14].

## 2. Material and Methods

We conducted a prospective, observational study in a group of adolescents with a diagnosis of T1D based on International Society for Pediatric and Adolescent Diabetes (ISPAD) criteria, with insulin treatment. For this study, 102 consecutive patients with type 1 diabetes, and without any signs and symptoms of diabetic neuropathy, were recruited. All adolescents were around 18 years of age, thus just before the transmission from pediatric to adult medical care (47 males and 55 females, mean age 17 years and 8 months). All children in the study were of European descent, and all of them used insulin analogs. In the group, 24 children were treated using functional intensive insulin therapy using pens, and 78 children were treated by insulin pumps. Patients’ demographic characteristics are presented in Table 1. Patients were treated at the Clinic of Endocrinology and Diabetology of The Children’s Memorial Health Institute in Warsaw. The study was performed as a part of the patients’ standard examination. Inclusion criteria were: type 1 diabetes according to ISPAD criteria [15], no signs or symptoms of diabetic neuropathy, no significant abnormalities in nerve conduction studies, and no diabetic ketoacidosis in the last 3 months. The exclusion criteria were: any signs of feet abnormality in clinical examination or history of congenital feet deformity, as well as any mobility or walking problems caused by musculoskeletal or nervous system diseases, status post big traumas, and fractures of the legs.

The study was approved by the Bioethics Committee of The Children’s Memorial Health Institute in Warsaw and followed the tenets of the Declaration of Helsinki. Written informed consent had been obtained from the patient’s legal guardians and from all the patients, after a detailed explanation of the nature of the non-invasive study before the tests started.

**Figure 1:**
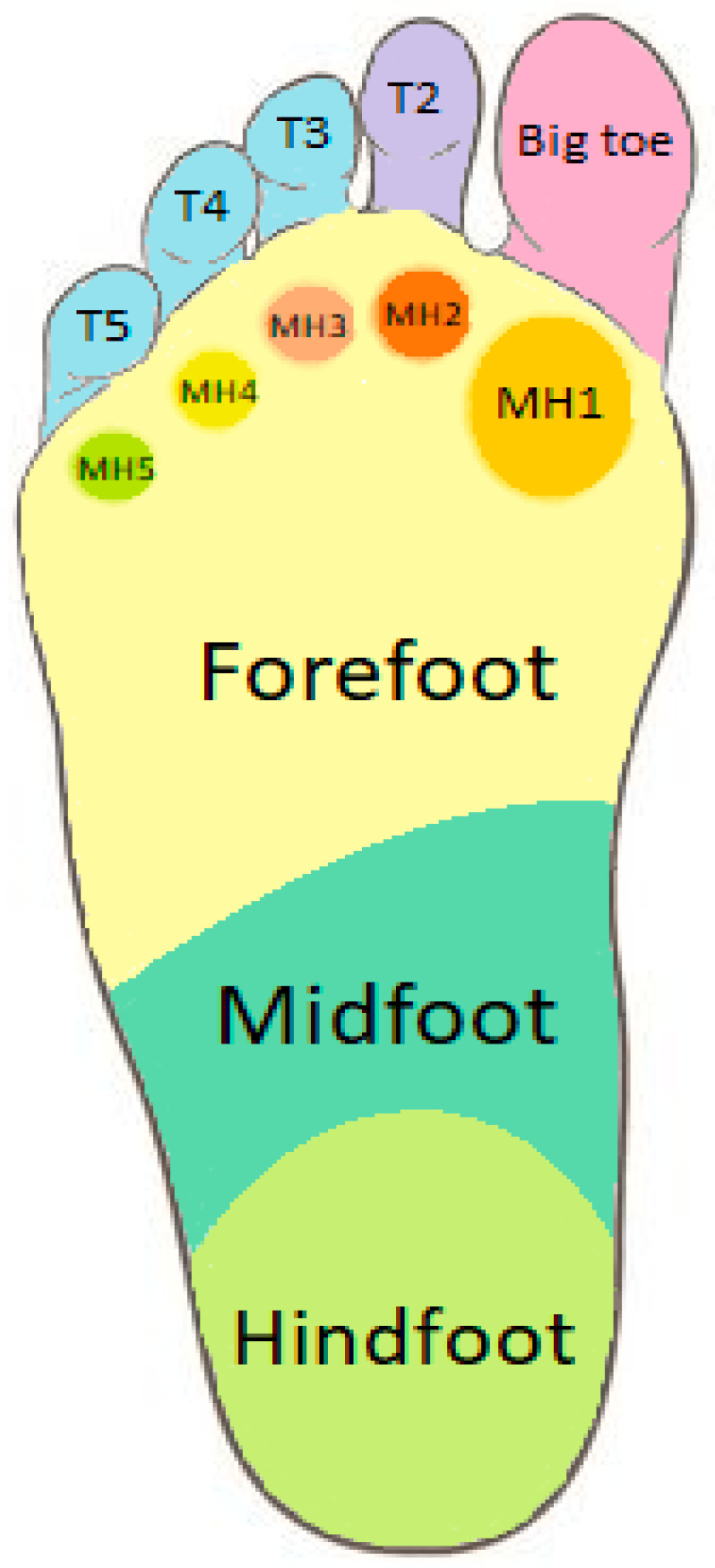
Illustrative drawing of foot division to analyzed areas.

Pedography was captured using a commercial Novel emed-X system (4 sensors per cm^2^, frequency 100 Hz, software v.23). Data of three left and three right footprints were captured during gait and later averaged. Footprints were analyzed in total foot and in the following areas (masks): hindfoot, midfoot, metatarsal bone 1 (MH1), metatarsal bone 2 (MH2), metatarsal bone 3 (MH3), metatarsal bone 4 (MH4), metatarsal bone 5 (MH5) or MH1-5 summarized as forefoot, big toe, 2 toe, 3–5 toes (345Toes), automatically identified by the Novel software version 23.3.36 (illustrative drawing is presented on Figure 1).

**Figure 2:**
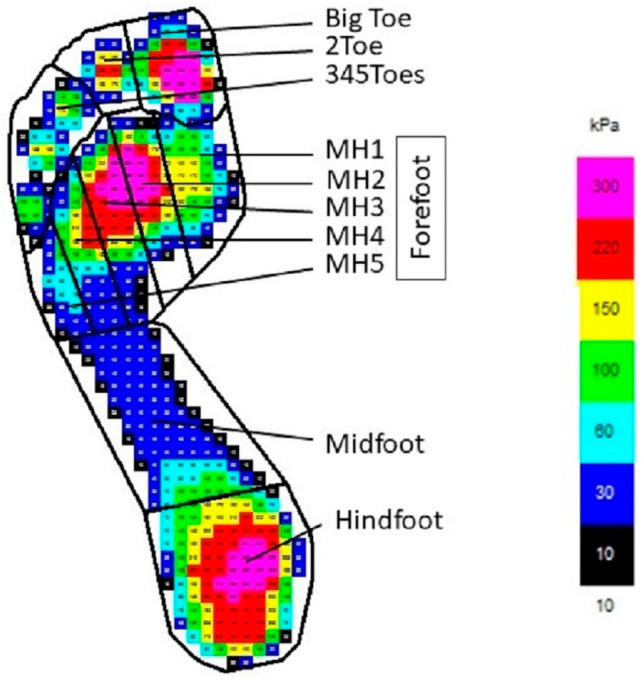
The automatic division of the footprint performed by the Novel software automasking tool.

The emed automasking tool follows the below-described rules (see Figure 2):

- The separation of straight lines between hindfoot and midfoot and midfoot and forefoot are perpendicular to the straight line that is created using the least square method;
- The boundary between the hindfoot and the midfoot is defined as 73% of the foot length from the top of the footprint;
- The boundary between the midfoot and the forefoot is defined as 45% of the foot length from the top;
- The boundary between the forefoot and toes and also between toes is defined considering the values of peak pressure under the toes and the gradients of pressure around these maximum values;
- The angles that define the metatarsal heads are defined as, respectively, 30%, 17%, 17%, 17%, and 19% of the long plantar angle for MH1, MH2, MH3, MH4, and MH5;
- The boundary between toes and the forefoot is defined as 10% of the foot length from the top.

Figure 2 presents an example of emed automasking of the footprint.

A number of parameters were calculated in Novel-projects software v. 23.3.36 describing pressure, force, and contact area distribution:

- Peak pressure (PP) [kPa]—the maximum pressure value on the total foot or area;
- Mean pressure (MP) [kPa]—the ratio of the sum of peak pressures over the sensors to the number of loaded sensors;
- Contact area (CA) [cm^2^] which is defined as the maximum contact area during stance [16],
- Force time integral [Ns]—the sum of products of force in each frame and the duration of one frame and represents the area under the force curve in time. Force is calculated as a sum of products of averaged pressure beneath the sensor and area of this sensor;
- Force time integral normalized to body weight [Ns/kg] as above but normalized to patient’s body weight;
- Pressure time integral [kPas]—the sum of products of peak pressure in each frame and the duration of one frame and represents the area under the pressure curve in time.

Those parameters were most commonly used in the literature as predictors of ulcers [13]. Patients’ results were compared to build-in reference data for grown-up subjects distributed by Novel GMBH with the system. Due to this, the studied T1D adolescents could be compared to what is known about pedography in diabetes in the literature. The actual state of the art is based on an older population. Such an approach allowed for pointing out possible pathologies in the studied group.

### Statistical Analysis

The data were described by mean, standard deviation, and minimal and maximum values. The data of study group were compared to means and standard deviations of reference ones using the test of differences between two means in independent measures (single direction condition) using TIBCO Software Inc. (2017) Statistica version 13 TIBCO Company. A level *p* < 0.05 was recognized as statistically significant.

## 3. Results

The results of the study are presented in Table 2 and Table 3. The orange color marks the results that are higher than the references; the green color marks the results that are lower than the references. The parameters in which statistically significant differences were found are marked in blue.

A statistically significant reduction in the contact area of the total foot, as well as areas MH5 and 345 toes, was found. In both feet, the peak pressure was also increased under the total foot, under hindfoot, midfoot, MH1, big toe, and 2 toe, although insignificantly. Statistically significant increases in mean plantar pressure in both lower limbs across the total foot, in the hindfoot, midfoot, and under MH1 were observed. In the left foot, the pressure under the big toe and 2 toe was also significantly increased. In addition, pressures under both forefeet, MH2 and MH3, big toes, and 2 toes were higher, but not statistically significantly. There was no pressure offloading under MH4 and MH5 and 345 toes of the left foot, but there was an overload of these areas in the right foot.

The integral parameters were presented in Table 3. They were limited in all areas, and most of them significantly.

## 4. Discussion

The results of this study revealed a significant reduction in the pressure time integral in the MH2 area, force time integral under the big toe, and pressure time integral in the MH4 area in both feet or at least one foot (Table 3). Additionally, peak pressure in the big toe area, peak pressure in MH5, and peak pressure in MH4 were normal (Table 2). These parameters are predictors of possible ulcers [13]. As participants of this study were functionally efficient—they walked at a normal speed, had a proper stance and swing phases during gait, and presented normal BMI—the feet’s exposition into abnormal pressure was limited in time. This indicates the limited probability of incoming ulcers and amputations so common in older diabetic patients. However, in the revision study Fernando et al. [17], they indicated the lack of thresholds of such parameters in the literature and proved the negative influence of neuropathy on ulcer risk. Therefore, the obtained results do not prejudge that there is no need to control integral parameters in assessments of risk of ulcers and possible amputations in the population of teenagers with T1D.

The analysis of the contact area revealed significantly decreased values in MH5, and simultaneously the mean pressure in MH5 was normal and in MH1 was significantly increased (Table 2). These findings suggest the tendency to unload MH5, which caused MH1 overloading. This was also observed in the study of older patients with T2D, who presented higher pressure in the medial forefoot, especially in subjects without neuropathy [18]. As the process of injury in diabetic feet is very likely to be initiated not on the skin surface, but in deeper tissue layers, the MH1 area should be carefully observed [19]. However, Sutkowska et al. [3] pointed to overload in the middle area of the forefoot in older patients with a BMI < 35. This may suggest the changes in the transverse arch of the forefoot that appear with age. Thus, rehabilitation applying exercises modeling feet shape could have an important preventive influence, and not only passive care with shoe inserts [20].

One of the findings was an increase in mean pressure in the big toe only in the left foot, but in the hind area in both feet. Most often, ulcer locations are in the big toe (51%) and hindfoot (29%) [21]. In the big toe area, Novel’s reference data differ between legs significantly (in right are higher), which caused those similar values of mean pressure in the left and right legs in the study group to reveal opposite statistical significance. However, the hindfoot area should be also monitored in young patients with T1D as its ulcers may implicate whole foot amputation.

This study also revealed overload in midfoot in mean pressure. The most important factor of peak pressure increases in midfoot was the presence of Charcot foot deformity [22]. Thus, future studies of feet geometry in young adult patients with T1D seems to be recommended.

Waldecker studied peak pressure, forces and integral pressure and forces parameters to distinguish ulcers causes [13]. Pedography studies use various pressure parameters, which differ in calculations of pressure, and sometimes also mean pressure is taken into consideration. Our study pointed to the need to analyze mean pressure in studies of young T1Ds, as it was increased in our study group, while peak pressure was preserved.

There are a number of publications describing incorrect plantar pressure distribution and its correlation with diabetic neuropathy and foot ulcer formation. It is estimated that up to 34% of diabetic patients will develop a foot ulcer during their lifetime [23,24]. More than four out of five lower limb amputations in diabetics are preceded by ulceration [8]. Defined risk factors for the development of ulcers include peripheral neuropathy, angiopathy, limited joint mobility, deformities, increased foot pressures, and minor traumas. Not all of them are avoidable, but it is possible to implement preventive measures and introduce screening allowing for their early detection. One such screening method is measuring plantar pressure distribution [23,25]. Forefoot structural deformities are prevalent in diabetes groups [26,27] increasing shoe plantar pressure at the metatarsal heads. The importance of footwear and insoles in offloading pressure for preventing plantar forefoot ulceration is well documented [28,29]. The authors studying pedographic abnormalities in diabetic patients either focus mainly on patients with type 2 diabetes, or do not divide patients into type 1 and type 2 diabetes. Moreover, all found in literature studies concerning this topic were performed on groups of middle-aged and elderly adults with diabetes [2,17,30,31]. This is understandable since the average age of diagnosis of diabetic foot is approximately 60 years of age [32]. Long disease duration is also required for the development of neuropathic and angiopathic complications leading to diabetic foot syndrome, so the risk of major amputations increases with age [33]. Therefore, the advanced development of diabetic complications is not present in pediatric patients. However, the purpose of this study was to investigate whether changes in the architecture of the foot and abnormalities of its contact with the ground in diabetic juvenile patients do not appear earlier. Detection of the early signs of such abnormalities is important as at that time correct prophylaxis, simple and inexpensive, can be introduced to prevent disabling abnormalities.

The results of this study show that the changes occur already in patients at the beginning of adulthood, not due to direct diabetes complications, but probably as the effect of not proper posture correction in childhood, bad shoes, or maybe undiagnosed neurological disturbances, as well as changed gait pattern, in the adolescent population which has the lowest mobility in human history. An interesting question is when in the pediatric population these abnormalities developed. In a big group of healthy children 7–12 years old, Feka et al. found significant differences in load distribution [34].

In our study, one of the exclusion criteria was the presence of neurological problems. However, we observed that in both feet, the peak pressure was increased under the total foot, under hindfoot, midfoot, MH1, big toe, and 2 toe. In the left foot, increased peak pressure was recorded under the forefoot and MH2, while in the right foot, pressure was recorded under the MH4 and MH5, and under the 345 toes. Changes in plantar pressure with respect to the reference data were not statistically significantly different. No high point pressure on individual areas of the foot was found, which is the most important pedographic risk factor in the development of corns and calluses putting added pressure on the underlying soft tissue [35,36] and leading to ulcers [37]. However, the results showed a decrease in the contact area of the foot with the ground and an increase in the mean pressure on these areas. Based on this finding it can be allowed for the hypothesis that such a trend could result in the development of increased point pressure in the future. The obtained results indicate the beginning of the development of excessive foot loads, which are different than those reported in adult diabetic patients [7].

The average duration of diabetes in this study was 7.36 years. This is a short period of life, and the impact of metabolic and vascular influences will significantly increase in time. Some of the examined patients developed diabetes in early childhood, so metabolic disorders occurred during the period of intense growth and formation of the musculoskeletal system. The abnormalities that develop during this time will stay with these patients for life. Additionally, Ilks et al. [38] showed that age has a significant influence on the progression of the pedographic changes, which is another argument for starting prevention in adolescents, as with early prophylaxis better results can be achieved.

According to the literature, the main reason for changed plantar pressure distribution is the loss of protective peripheral sensation resulting from diabetic peripheral neuropathy, leading to high, long-lasting pressure on the loaded plantar surface [7]. The pathology of motor nerves causes muscle weakness and changes in the flexor-extensor force ratio, which leads to foot deformation. Therefore, there is a need to extend further research on plantar pressure distribution in comparison to the results of electromyography (EMG) and nerve conduction studies (NCS).

To the authors’ knowledge, this is the first study on plantar pressure distribution in a pediatric population, adolescents, only with type 1 diabetes. The obtained results are important for screening purposes, as they can be used as reference data for clinicians assessing the feet of adolescent patients with T1D.

The limitation of this study is the lack of a control group of healthy peers, but due to requirements for building such a control group—with perfectly healthy locomotion structures, and without perinatal or neonatal disorders history, any traumas, any surgeries, and additionally a group matched in age—building such a group was extremely difficult. From the authors’ practice, using normative data from the producer base allows for better comparisons to the results in other studies.

## 5. Conclusions

This study showed that T1D patients of age near adulthood without signs and symptoms of neuropathy do not present typically for T2D patients feet pressure distribution regarded as predestining to the ulcers risks. However, increased values in mean pressure and reduced contact area in T1D patients insist on screening in clinical practice. The obtained values of our study group may be used as references for such a screening. Patients with T1D may require pedographic control tests and then if abnormalities in foot plantar pressure distribution exist, prevention measures should be implemented, and the pressure distribution and its possible changes should be monitored in time. 

In this study, pedography parameters of younger T1D patients were analyzed in comparison with the references of older ones. The older patients are deeply analyzed, the relations between pedography and clinics were assessed, and the risk factors of comorbidities were revealed. As this study opens the topic of pedography in young T1Ds, such an approach allowed us to screen the data of this study on the presence of pathology revealed in older patients [33].

These preliminary results point to the need for further research and analysis which should include various risk factors such as foot anatomy, body posture, or certain metabolic factors. 

## Figures and Tables

**Table 1:** Demographic characteristics of the patients.

	Mean ± SD	Min–Max
Age (years)	17.8 ± 0.14	17.3–18.6
Age at diabetes onset	10.3 ± 4.2	1.8–17.0
Diabetes duration	7.36 ± 4.2	0.8–16.1
HbA1c for whole diabetes duration	8.1 ± 1.2	6.2–12.1
BMI	23.3 ± 3.3	17.3–32.7

**Table 2:** The comparison of study group and reference data of peak pressure, mean pressure, and contact area. *p*-value was calculated in tests of differences between two means in independent measures (single direction condition).

Peak Pressure [kPa] LEFT FOOT						Peak Pressure [kPa] RIGHT FOOT					
**Foot Area**	Result	±SD	*p*-Value	References	±SD	Foot Area	Result	±SD	*p*-Value	References	±SD
Totalfoot	595.16	203.0	0.25	548.9	195.1	Totalfoot	577.53	195.3	0.35	539.9	118.1
Hindfoot	345.79	74.7	0.45	345.1	92.2	Hindfoot	339.8	65.7	0.2	322.5	1.5
Midfoot	124.52	44.1	0.23	113.6	45.7	Midfoot	128.19	38.9	0.13	115.6	80.3
Forefoot	459.91	180.5	0.75	448.40	150.0	Forefoot	427.71	155.8	0.54	447.4	1.2
MH1	265.13	126.9	0.39	242.3	139.0	MH1	256.62	115.9	0.9	250.7	121.8
MH2	361.06	141.7	0.23	348.7	103.8	MH2	349.59	105.8	0.2	379.6	1.0
MH3	336.49	93.3	0.7	341.5	103.3	MH3	334.7	105.8	0.3567	355.5	225.6
MH4	260.32	91.9	0.49	273.4	90.9	MH4	270.38	118.8	0.2	250	1.8
MH5	237.40	167.3	0.58	254.6	178.8	MH5	226.72	146.7	0.52	207	83.3
BigToe	449.61	225.3	0.14	380.5	245.2	BigToe	443.82	235.6	0.6	420	1.3
2Toe	179.30	95.8	0.83	174.1	92.4	2 Toe	184.31	99.6	0.43	168.9	68.3
345Toes	121.44	65.4	0.33	129.8	82.4	345 Toes	134.85	92.5	0.3	116.1	2.6
**Mean Pressure [kPa] LEFT FOOT**						**Mean Pressure [kPa] RIGHT FOOT**					
**Foot Area**	**Result**	**±SD**	***p*-Value**	**References**	**±SD**	**Foot Area**	**Result**	**±SD**	***p*-Value**	**References**	**±SD**
Totalfoot	133.34	14.4	0	117.88	10.7	Totalfoot	132.58	14.2	0	118.66	10.7
Hindfoot	183.37	27.5	0	159.7	20.7	Hindfoot	181.16	24.3	0	159.14	21.5
Midfoot	57.77	18.0	0.03	50.15	18.3	Midfoot	59.17	17.9	0.019	50.4	17.7
Forefoot	146.25	21.8	0.11	139.18	19.0	Forefoot	145.43	22.6	0.07	137.52	16.9
MH1	124.62	34.0	0.0028	101.72	43.5	MH1	123.62	32.0	0.029	108.38	38.0
MH2	181.18	37.8	0.0945	168.06	36.9	MH2	178.64	34.2	0.3	178.16	42.2
MH3	171.56	34.4	0.25	167.37	34.5	MH3	171.59	34.4	0.17	167.25	34.0
MH4	129.51	36.0	0.28	137.71	39.7	MH4	132.63	35.7	0.9	124.76	34.0
MH5	99.86	43.4	0.45	106.98	49.9	MH5	98.42	39.3	0.32	90.69	39.2
BigToe	142.75	42.9	0.0136	120.07	47.1	BigToe	143.02	45.7	0.29	133.16	45.1
2Toe	73.34	24.2	0.05	69.83	28.7	2 Toe	75.09	25.6	0.41	70.69	26.8
345Toes	47.50	17.1	0.48	48.69	22.3	345 Toes	49.82	20.3	0.23	44.84	18.4
**Contact Area [cm^2^] LEFT FOOT**						**Contact Area [cm^2^] RIGHT FOOT**					
**Foot Area**	**Result**	**±SD**	***p*-Value**	**References**	**±SD**	**Foot Area**	**Result**	**±SD**	***p*-Value**	**References**	**±SD**
Totalfoot	126.42	17.7	0.01	135	14.9	Totalfoot	128.00	18.3	0.05	135.03	14.2
Hindfoot	33.12	4.4	0.33	33.97	4.0	Hindfoot	33.16	4.4	0.14	34.37	3.8
Midfoot	24.21	7.5	0.09	27.25	4.1	Midfoot	24.6	7.3	0.06	27.13	3.6
Forefoot	49.10	6.2	0.25	50.57	6.3	Forefoot	49.46	6.1	0.28	50.79	5.4
MH1	12.83	2.2	0.28	12.48	2.4	MH1	12.87	1.9	0.87	12.99	2.1
MH2	10.13	1.6	0.52	10.45	1.8	MH2	10.26	1.6	0.75	10.3	1.4
MH3	11.13	1.4	0.12	11.67	1.6	MH3	11.03	1.5	0.37	11.5	1.5
MH4	9.29	1.2	0.14	9.61	1.3	MH4	9.3	1.2	0.77	9.68	1.2
MH5	5.72	0.9	0.0004	6.35	1.1	MH5	5.79	0.9	0.0086	6.3	1.0
BigToe	10.45	1.8	0.1681	10.95	1.7	BigToe	10.54	2.2	0.074	11.33	1.8
2Toe	3.53	0.9	0	4.42	1.2	2 Toe	3.67	1.0	0.008	4.25	1.3
345Toes	5.94	2.4	0.0005	7.68	2.2	345 Toes	6.48	2.7	0.26	7.11	2.6

**Table 3:** The comparison of study group and reference data of force time integral, force time integral normalized to body weight, and pressure time integral. *p*-Value was calculated in tests of differences between two means in independent measures (single direction condition).

Force Time Integral [Ns] LEFT FOOT						Force Time Integral [Ns] RIGHT FOOT					
**Foot Area**	Result	±SD	*p*-Value	References	±SD	Foot Area	Result	±SD	*p*-Value	References	±SD
Totalfoot	390.9	75.2	0	490.1	86.2	Totalfoot	391.2	75.1	0	485.1	88.9
Hindfoot	113.7	30.0	0	152.0	37.8	Hindfoot	106.2	23.0	0	147.3	33.9
Midfoot	33.7	20.3	0.1840	38.2	20.9	Midfoot	34.8	20.3	0.3410	36.8	17.9
Forefoot	203.7	45.5	0.0002	246.0	60.2	Forefoot	208.5	47.9	0.0022	242.8	50.8
MH1	43.9	17.8	0.4412	44.6	26.1	MH1	44.2	16.5	0.1655	48.5	24.8
MH2	50.5	13.1	0.0160	61.0	19.4	MH2	51.4	11.9	0.0001	63.8	19.3
MH3	55.1	14.1	0.0001	69.6	21.8	MH3	57.0	15.0	0.0023	68.1	19.2
MH4	37.1	12. 5	0.0008	47.8	18.5	MH4	38.7	13.4	0.0896	43.2	14.9
MH5	17.1	8.8	0.0101	22.9	15.3	MH5	17.2	9.1	0.1950	19.2	11.4
BigToe	29.5	14.4	0.0695	35.2	21.3	BigToe	30.2	15.5	0.0055	41.8	29.5
2Toe	4.8	2.5	0	7.9	5.3	2 Toe	5.1	2.9	0.0008	7.7	5.0
345Toes	5.5	4.3	0.0001	10.7	10.0	345 Toes	6.4	5.8	0.0624	8,7	7,5
**Force Time Integral Normalized to Body Weight** **[Ns/kg] LEFT FOOT**						**Force Time Integral Normalized to Body Weight** **[Ns/kg] RIGHT FOOT**					
**Foot Area**	**Result**	**±SD**	***p*-Value**	**References**	**±SD**	**Foot Area**	**Result**	**±SD**	***p*-Value**	**References**	**±SD**
Totalfoot	57.7	4.4	0	75.6	9.9	Totalfoot	57.7	4.5	0	74.5	10.3
Hindfoot	16.9	3.7	0	23.5	5.4	Hindfoot	15.8	2.9	0	22.6	4.5
Midfoot	4.8	2.6	0.0468	5.9	3.0	Midfoot	5.0	2.5	0.1315	5.7	2.8
Forefoot	30.0	4.2	0	37.9	7.7	Forefoot	30.7	4.4	0	37.3	6.8
MH1	6.5	2.3	0.2660	6.9	3.9	MH1	6.5	2.1	0.0450	7.5	3.6
MH2	7.5	1.6	0	9.4	2.6	MH2	7.6	1.3	0	9.8	2.9
MH3	8.1	1.6	0	10.7	2.9	MH3	8.4	1.6	0	10.5	2.8
MH4	5.5	1.5	0	7.4	2.7	MH4	5.7	1.6	0.0154	6.6	2.1
MH5	2.5	1.1	0.0009	3.6	2.5	MH5	2.5	1.2	0.0605	3.0	1.8
BigToe	4.4	2.1	0.0378	5.4	3.1	BigToe	4.4	2.2	0.0015	6.3	4.0
2Toe	0.7	0.4	0.0001	1.2	0.9	2 Toe	0.8	0.5	0.0020	1.2	0.8
345Toes	0.8	0.7	0.0001	1.7	1.8	345 Toes	1.0	1.0	0.1226	1.3	1.3
**Pressure Time Integral [kPas] LEFT FOOT**						**Pressure Time Integral [kPas] RIGHT FOOT**					
**Foot Area**	**Result**	**±SD**	***p*-Value**	**References**	**±SD**	**Foot Area**	**Result**	**±SD**	***p*-Value**	**References**	**±SD**
Totalfoot	219.16	54.16	0.0001	274.99	81.16	Totalfoot	212.90	52.27	0	269.70	80.90
Hindfoot	79.44	19.29	0	108.63	28.52	Hindfoot	74.04	14.78	0	101.09	24.27
Midfoot	37.79	14.26	0.0599	43.59	19.34	Midfoot	38.40	13.58	0.1858	41.51	17.20
Forefoot	133.92	44.82	0.0011	171.74	70.31	Forefoot	131.30	47.25	0.0110	169.46	63.16
MH1	76.49	33.67	0.0890	89.28	58.79	MH1	76.00	33.97	0.0535	91.09	55.23
MH2	101.24	27.08	0.0007	124.86	39.64	MH2	100.96	24.00	0	135.72	50.60
MH3	101.42	28.81	0.0003	128.37	41.72	MH3	103.18	32.88	0.0003	134.12	47.60
MH4	83.23	30.27	0.0028	105.36	41.09	MH4	88.11	38.48	0.2219	95.15	31.94
MH5	69.83	41.00	0.0414	90.27	74.78	MH5	69.66	41.73	0.3659	73.28	50.12
BigToe	101.46	59.67	0.1886	115.51	88.11	BigToe	99.89	57.79	0.0326	129.14	92.21
2Toe	36.47	18.41	0.1230	47.94	30.02	2 Toe	37.50	21.49	0.0506	46.76	29.70
345Toes	27.81	17.25	0.0077	40.60	36.06	345 Toes	30.34	23.15	0.1928	35.41	27.40

## Data Availability

The anonymised data could be obtained on the request to the authors.
